# Source-Related Effects of Wastewater on Transcription Factor (AhR, CAR and PXR)-Mediated Induction of Gene Expression in Cultured Rat Hepatocytes and Their Association with the Prevalence of Antimicrobial-Resistant *Escherichia coli*

**DOI:** 10.1371/journal.pone.0138391

**Published:** 2015-09-18

**Authors:** Keerthi S. Guruge, Noriko Yamanaka, Miyuki Sonobe, Wataru Fujizono, Miyako Yoshioka, Masato Akiba, Takehisa Yamamoto, Derrick I. Joshua, Keshava Balakrishna, Nobuyoshi Yamashita, Kurunthachalam Kannan, Toshiyuki Tsutsui

**Affiliations:** 1 Pathology and Pathophysiology Research Division, National Institute of Animal Health, National Agriculture and Food Research Organization, Tsukuba, Ibaraki, Japan; 2 Central Livestock Hygiene Service Center, Wakakusu, Saga, Japan; 3 Central Livestock Hygiene Service Center, Jonan, Minami-Ku, Kumamoto, Japan; 4 Bacterial and Parasitic Disease Research Division, National Institute of Animal Health, National Agriculture and Food Research Organization, Tsukuba, Ibaraki, Japan; 5 Viral Diseases and Epidemiology Research Division, National Institute of Animal Health, National Agriculture and Food Research Organization, Tsukuba, Ibaraki, Japan; 6 Department of Civil Engineering, Manipal Institute of Technology, Manipal University, Manipal, Karnataka, India; 7 National Institute of Advanced Industrial Science and Technology, Tsukuba, Ibaraki, Japan; 8 Wadsworth Center, New York State Department of Health and Department of Environmental Health Sciences, State University of New York at Albany, Albany, New York, United States of America; University of Florida, UNITED STATES

## Abstract

Extracts of wastewater collected from 4 sewage treatment plants (STPs) receiving effluents from different sources in South India were investigated for their levels of transcription factor-mediated gene induction in primary cultured rat hepatocytes. In addition, the relation between gene induction levels and the prevalence of antimicrobial-resistant *Escherichia coli* (*E*. *coli*) in wastewater was examined. STP-3, which treats only hospital wastewater, exhibited significantly greater induction potency of all 6 drug metabolizing cytochrome P450 (CYP) genes examined, CYP1A1, 1A2, 1B1, 2B15, 3A1, and 3A2, whereas the wastewater at STP-1, which exclusively receives domestic sewage, showed significantly diminished levels of induction of 3 CYP genes when compared to the levels of CYP induction at STP-2, which receives mixed wastewater. Samples collected during the monsoon season showed a significantly altered gene induction capacity compared to that of samples from the pre-monsoon period. The data suggest that the toxicity of wastewater in STPs was not significantly diminished during the treatment process. The chemical-gene interaction data predicted that a vast number of chemicals present in the wastewater would stimulate the genes studied in the rat hepatocytes. The multivariable logistic regression analysis demonstrated that the prevalence of isolates resistant to cefotaxime, imipenem and streptomycin was significantly correlated with the levels of induction of at least three CYP-isozymes in STP wastewater. In addition, the resistance of isolates in treatment plants was not altered by the treatment steps, whereas the sampling season did have an impact on the resistance to specific antimicrobials. The identification of receptor-mediated gene regulation capacities offers important data not limited to the (synergistic) physiological role of chemicals in biological systems but may provide new insight into the link between the effects of known/unknown drugs and prevalence of antimicrobial-resistant bacteria in wastewater.

## Introduction

Sewage treatment plants (STPs) often receive wastewater originating from households, hospitals, industries, surface runoff and wet deposition, thereby forming a complex mixture of wastes. STPs play an important role in the collection, treatment, and dissemination of a vast amount of anthropogenic chemicals and biological wastes. The extensive monitoring studies of wastewater from STPs have revealed that they contain a diverse range of micropollutants, including persistent organic pollutants (POPs), polycyclic aromatic hydrocarbons (PAHs), flame retardants, pesticides, perfluorinated compounds, pharmaceuticals, personal care products (PPCPs) and illicit drugs [1–7].

STP wastewaters can also release antimicrobial-resistant bacteria (ARB) into the environment, which is a growing public health threat in terms of the dissemination of superbugs [8, 9]. It has been reported that a novel β-lactam drug-resistance gene, New Delhi metallo-β-lactamase 1 (NDM-1), originated in India [10]. A broad-spectrum antimicrobial-resistant *Enterobacteriaceae* harboring NDM-1 has been repeatedly detected in the Indian environment, and the propagation of NDM-1 genes in municipal wastewater has been reported in China [11, 12]. In our previous study, we found for the first time that hospital wastewater has a strong impact on the prevalence of antimicrobial-resistant *E*. *coli* in Indian STPs [13]. Although investigations are being carried out on the dissemination of ARB in India, assessment of the micropollutant-related toxicological potency of wastewater has received less attention.

Wastewater may by inherently toxic due to the presence of complex chemical mixtures and thus may mimic diverse modes of action in biological systems. However, instrumental analytical techniques alone may provide little or no information on the potential biological effects of complex environmental mixtures [14, 15]. Over the years, cell-based *in vitro* bioassays have been extensively employed as a tool to investigate the biological and toxicological effects of individual or admixed chemicals [16–19]. Although cell bioassays and instrumental analyses are different tools, they are closely analogous and can provide comparable, but complementary, results with regard to the relative amounts of certain chemical groups [20–22]. The bioassays may not be specific in detecting the chemical identity of toxicants in the sample, but batteries of *in vitro* reporter gene assays based on diverse receptor-mediated actions are widely used to evaluate the treatment performance together with toxic profiles of wastewater effluents [23–27]. In addition to recombinant reporter gene assays, primary cultured mammalian hepatocytes have been widely employed to study the effects of a single chemical or chemical mixtures by measuring the induction of drug-metabolizing enzymes [28–30]. Moreover, the treatment of hepatocytes with xenobiotics may reveal toxicogenomic effects—such as the alteration of receptor-mediated gene expressions—that would be useful for predicting biological effects at the whole animal level [31]. In particular, the aryl hydrocarbon receptor (AhR)- and pregnane X receptor (PXR)-mediated endpoints should be considered for water quality assessments because most chemicals found in wastewater could pose health-related responses mediated through these two intercellular receptors [32]. However, there have been few or no studies on biological effects confirmed by *in vitro* gene-expression assay of wastewater.

In light of our previous findings revealing that hospital wastewater has a major impact on the dissemination of antimicrobial-resistant *E*. *coli* in STPs in India, we further investigated the toxic potentials of chemical substances in the STP environment together with the ecological risks that they may pose by distinguishing associations among toxic effects and the nature of the ARB diversity. First, we report the results of the AhR-, constitutive androstane receptor (CAR)- and PXR-mediated gene expression potentials of wastewater collected from 4 STPs located in South India, by measuring the levels of induction of selected genes, i.e., drug metabolizing cytochrome P450 (CYP) 1A1, 1A2, 1B1, 2B15, 3A1 and 3a2, in rat hepatocytes cultured with the wastewater extracts. These gene expression data were used to evaluate the treatment performances and to determine the existence of micropollutants in STPs. Amos et al. [33] recently reported the importance of numerous chemical and physical parameters in addition to antimicrobials in river water in an effort to elucidate the factors and mechanisms that regulate the environmental resistome. To our knowledge, the effects of complex chemical mixtures in wastewater have not been integrated with other variables to determine their combined roles in the prevalence of ARB. We therefore investigated the associations among multiple environmental variables such as STP type, treatment step, climatic effect, gene regulation potency, and presence of antimicrobial-resistant *E*. *coli* in wastewater samples collected from STPs by performing multivariable logistic regression analysis.

## Materials and Methods

### Study area and sample collection

In the pre-monsoon and monsoon periods of 2013, water samples were collected in 1L polypropylene bottles from four STPs located in Karnataka State, South India. STP-1, STP-3 and STP-4 are municipal STPs receiving an average of 1800, 1800 and 1500 m^3^ of wastewater per day, respectively. The source of sewage in STP-1 is exclusively domestic wastewater from approximately 9000 inhabitants apart from academic institutions and offices. STP-2 and STP-4 receive a mixture of hospital and domestic wastewater and treat wastewater generated by approximately 60,000 residents, office and academic institutions, including a major hospital treating 1500 in-patients and 2300 out-patients per day. STP-3 receives approximately 50 m^3^ wastewater daily, and was built to treat the wastewater from a hospital serving approximately 100 in-patients and 400 out-patients daily. Samples were collected from each of four different treatment steps—i.e., the equalization (step 1), aeration (step 2), settling (step 3), and outlet (step 4) steps—at each STP except for the equalization step in the STP-3 due to the inability to access the sampling point. Wastewater samples used for testing antimicrobial resistance and for studying gene expression potency were collected at the same time. [13]. Labeling of the sites remains consistent with the previous manuscript [13] to avoid confusing of new data from the STP-4. Samples were collected in the morning and stored at -20°C after obtaining aliquots for bacterial isolation. Specific permissions were obtained from the hospital and university authorities that run these STPs and the purpose of the study was explained to them.

### Chemicals

Sodium salt of phenobarbital (PB), dexamethasone (DXM), and omeprazole (OMP) were purchased from Wako Pure Chemical Industries (Osaka, Japan). Rifampicin (RIF), 1,4-bis[2-(3,5-dichloropyridyloxy)]benzene (TCPOBOP) and β-naphthoflavone (b-NF) were from Sigma (St. Louis, MO). 2,3,7,8-tetrachlorodibenzo-*p*-dioxin (TCDD) and pregnenolone-16α-carbonitrile (PCN) were from Cambridge Isotope Laboratories (Andover, MA) and Enzo Life Sciences (Farmingdale, NY), respectively. Analytical grade dichloromethane, hexane, anhydrase NaSO_4_, dimethyl sulfoxide (DMSO) and all the antimicrobials were purchased from Wako Pure Chemical Industries (Osaka, Japan). All chemical supplements and media for bioassay were purchased from either Sigma (St. Louis, MO) or Gibco (Grand Island, CA).

### Preparation of wastewater extracts

Unfiltered wastewater samples (500 mL) in 1L glass separating funnels were extracted with a 50 mL dichloromethane and hexane mixture (1:2) by mechanical shaking for 15 min at 200 strokes/min. The supernatant (organic layer) was collected after samples were allowed to settle for 30 min and the extraction was repeated. Then, the two organic layers were combined, and concentrated at 40°C using a rotary evaporator followed by dehydration over anhydrous NaSO_4_. The extracts were further evaporated to near dryness by purging nitrogen at 40°C and re-dissolved in DMSO to prepare a final volume of 100 μl. Overall, the enrichment of chemicals from STP water into the DMSO extract was 5000 times.

### Cell culture

Primary culture of rat hepatocytes was prepared from 3 male Sprague-Dawley rats (age 7 weeks; average weight: 180 g; Japan SLC, Shizuoka, Japan). Prior to the experiment, the animals were assimilated for 7 days in a temperature (23±1°C), humidity (50±10%), and light (12-h dark/12-h light cycle) controlled facility with free access to laboratory chow (MF; Oriental Yeast Co., Ltd., Tokyo, Japan) and tap water. All experimental procedures were approved by the committee conferring to the National Institute of Animal Health (Tsukuba, Japan) guidelines for animal experimentation.

Hepatocytes were isolated by a two-step in situ perfusion via the portal vein, which was performed using a peristeric pump according to the previously described method with some modifications [34]. This technique can also be seen partially as a video protocol [35]. In brief, Ca^+2^/Mg^+2^-free Hank’s solution containing 0.05 mM ethylene glycol tetra acetic acid (EGTA) with 10 mM Hepes (37°C) was used for the pre-perfusion. Then collagenase digestion was performed with Ca^+2^/Mg^+2^-free Hank’s solution supplemented with 0.5 g/L collagenase, 0.05 g/L trypsin inhibitor and Hepes. The perfused liver cells were dispersed in the same perfusion solution, and then the adipose tissues and vessels were removed, and filtered with a 100 μm nylon cell strainer (Becton Dickinson and Co., Franklin Lakes, NJ). The cells were washed three times by low speed centrifugation (50 x g) with Ca^+2^/Mg^+2^-free Hank’s solution to remove non-parenchymal liver cells and re-suspended in cold serum-free and phenol red-free William’s E (WE) medium supplemented with a mixture of antibiotics, insulin, dexamethasone, aprotinin and a mixture of trace elements. The isolated hepatocytes had >90% purity and viability. Then, hepatocytes (1 x 10^6^/well) were seeded into a 6-well plastic dish with a positively charged surface (Primaria, Becton Dickinson and Co., Franklin Lakes, NJ) and maintained at 37°C under a humidified atmosphere of 5% CO_2_ in air for 24 h prior to dosing with test materials.

### Cell treatment

After 24 h of culture, the media were replaced and the cells were exposed to either prototypical CYP inducers or sample extracts. The inducers, dissolved in DMSO, were dosed at a quantity of either 10 μM β-NF, 16 x 10^−6^ μM TCDD, 20 μM PCN, 20 μM DXM, 10 μM RIF, 10 μM THPOBOP or 10 μM OMP. Phenobarbital was dissolved in WE medium and administered at 1.0 x 10^3^ μM. Vehicle and procedural blank controls contained 0.5% DMSO of the total media volume. Cell viability was assessed by morphological appearance and alamarBlue assay (AdB Serotec, Oxon, UK). The maximum concentration of the effluent that could be dosed into rat hepatocytes without inducing cytotoxicity (< 20%) was determined by treating diluted extracts in DMSO with cells prior to the actual assay. This final volume of effluent in the wells corresponded to 5 times the original concentration in wastewater. Exposures were carried out for 48 h with a renewal of the media and analytes after 24 h. All the chemicals and sample extracts were kept at -20°C and assayed in triplicate.

### RNA extraction and gene expression analysis

Total RNA was isolated from hepatocytes using a QuickGene RNA cultured cell kit and QuickGene-810 Nuclear Acid Isolation System (Fuji Film, Japan) with Cell-Plus mode according to the manufacturer’s instructions to avoid any contamination of the genomic DNA [29]. The RNA purity was determined by the optical densities measured at 260 and 280 nm (260/280 >1.9), and total RNA concentrations were determined by the absorbance at 260 nm. The RNA integrity was further estimated by the 28S and 18S rRNAs in 1% agarose gel electrophoresis.

The expression levels of selected genes encoding AhR, PXR and CAR were analyzed using a real-time qPCR (quantitative polymerase chain reaction) system (Mx3000P) with a Brilliant II SYBR^®^ Green QRT-PCR Master Mix Kit, 1-Step (Agilent Technologies, La Jolla, CA). The reagent mixture, primer concentration and experimental protocol have been described elsewhere [36]. Briefly, 12.5 μL of SYBR Green master mix, 0.5 μL each of the upstream and downstream primer (5 pmol/μL), 0.375 μL of diluted ROX reference dye, and 1.0 μL of RT/RNase block enzyme mixture were well mixed and adjusted to 23 μL with nuclease-free water. A 200 ng aliquot of the isolated total RNA sample (2 μL) was added to the reagent mixture described above to obtain a total volume of 25 μL. The qPCR instrument was programed with two-step cycling for DNA synthesis followed by a melting curve analysis using the following protocol: 30 min at 50°C (for reverse transcription) and 10 min at 95°C (denaturing), followed by 40 cycles of 95°C for 30 s, 55°C for 1 min, and 72°C for 30 s for amplification with a fluorescence measurement at the end of the last step. The melting curve was programmed as follows: 95°C for 30 s, 55°C for 30 s followed by 55–95°C at a heating rate of 0.5°C/s together with continuous fluorescence measurement.

The primers for target genes were either designed by us and synthesized at Hokkaido System Science (Sapporo, Japan) or purchased pre-designed once from Takara Bio (Otsu, Japan); all primers are listed in S1 Table. The relative expression of genes of interest was estimated by the delta–delta method with normalization to the glyceraldehyde 3-phosphate dehydrogenase (GAPDH) gene as the control gene. The analyses of differences in gene expression levels among extracts or standards were carried out by normalizing the expressions to the corresponding vehicle controls. Chemicals which showed associations with targeted genes for rats were retrieved from the Comparative Toxicogenomics Database [37].

### Isolation of bacteria and antimicrobial susceptibility testing

We employed our previously available antimicrobial resistance data for 160 *E*. *coli* isolates from 3 STPs [STP-1 (50 isolates), STP-2 (54 isolates) and STP-3 (56 isolates)] [13]. These isolates were randomly selected from pre-monsoon and monsoon wastewater samples and tested for antimicrobial susceptibility against 12 antimicrobials. The antimicrobials used in this study were ampicillin, cefazolin, cefoxitin, cefotaxime, imipenem, chloramphenicol, tetracycline, streptomycin, kanamycin, sulfamethoxazole-trimethoprim, nalidixic acid and ciprofloxacin. In addition, similar to our previous study, an additional 66 isolates which were collected from wastewater samples in STP-4 during the same season were tested for their antimicrobial susceptibility to study the associations between gene expression data and *E*. *coli* resistance to the antimicrobials. The details of the isolation of bacteria and disk diffusion test for antimicrobial susceptibility were reported elsewhere [13].

### Statistical analysis

For the statistical analysis, gene expression data were log-transformed, as these values are calculated as an exponent of two in the delta-delta method. The correlations among the levels of gene expression in STP wastewaters were determined by linear regression analysis. In addition, multivariable regression analysis was performed to investigate the relationships between gene expression as the target variable and explanatory variables such as the STP facility, treatment process, and season. For each explanatory variable, STP-2 (a relatively newly built facility that receives a mixture of domestic and hospital wastewater) as the STP facility, step 1 as the processing step and pre-monsoon as the season were used as reference levels to determine the significance. The *F*-tests were performed to test the statistical significance of the coefficients of each variable.

We further investigated the relationships between receptor-mediated gene expressions and the prevalence of antimicrobial-resistant *E*. *coil* isolates from the STPs by performing multivariable logistic regression analysis. Since the prevalence of resistance was measured once for each water sample while gene expression was measured in triplicate, the mean values for log-transformed gene expression data were assigned for each sample. The prevalence of resistance of the *E*.*coli* isolates to a selected antimicrobial was designated as the target variable to ascertain the association with the particular level of gene expression. In addition to the particular gene expression, potential confounding variables, i.e., the STP facility, treatment process, and season, were entered into the multivariable models as explanatory variables. The statistical significance of coefficients for each explanatory variable was tested by the Wald test. All statistical analyses were performed using the R software version 3.1.0 [38]. For all tests, results were considered statistically significant when P < 0.05.

## Results

### Induction of CYP genes by prototype inducers

**Fig 1:**
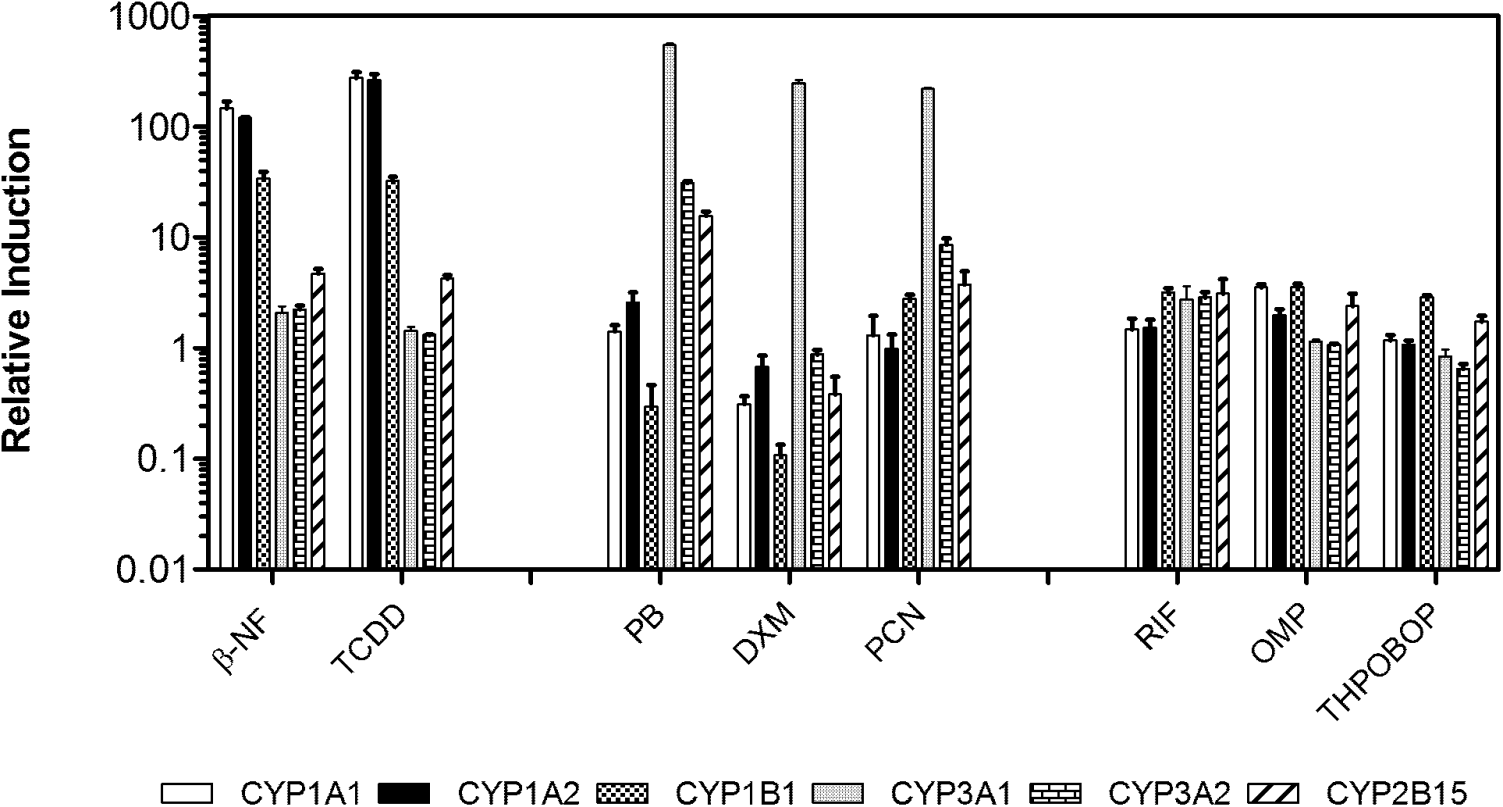
Comparison of CYP gene expression by the prototypical inducers in rat hepatocytes. Data are reported as means ± SD (standard deviation).

The vehicle-normalized relative expression levels of receptor-mediated CYP genes in rat primary hepatocytes treated with prototype inducers, which are often tested on mammalian cells, for 48 h are shown in Fig 1. The levels of CYP1A1, 1A2 and 1B1 expression induced by the AhR agonists’ β-NF and TCDD were 147±13, 121±1.3, 34±2.8 and 275±21, 263±20, 33±1.6, respectively. The inductions of CYP3A1 expression by the PXR agonists, PB, DXM and PCN, were quite strong, with values of 547±11, 247±10 and 219±3.8, respectively. Only PB and PCN showed higher inductions of CYP3A2 than other inducers, with mean values of 31±0.38 and 8.9±0.68, respectively. The PB treatment exhibited the highest level of induction of CAR-responsive CYP2B15, at 16±0.78, followed by PCN at 3.8±0.69. The RIF, OMP and THPOBOP did not show any capacity for the induction of either an AhR, CAR or PXR response in rat hepatocytes.

### Associations among CYP gene expression induced by STP wastewater

We examined correlations between all pairs of gene expression data to evaluate associations among gene induction potentials in rat hepatocytes treated with wastewater extracts. Interestingly, all 6 CYP genes studied demonstrated a significant positive correlation (r^2^: 0.547–0.954) (Table 1). The CYP1 genes showed the highest correlations among them at r^2^ values of 0.837 to 0.954, while the remaining genes had r^2^ values of 0.547 to 0.873. The CYP2B15 showed the smallest correlation with other genes (r^2^: 0.547–0.742).

**Table 1:** Correlation coefficients among CYP gene expression potentials in STP wastewater.

	CYP1A2	CYP1B1	CYP3A1	CYP3A2	CYP2B15
CYP1A1	0.954*	0.837*	0.847*	0.785*	0.668*
CYP1A2		0.841*	0.843*	0.784*	0.683*
CYP1B1			0.870*	0.910*	0.669*
CYP3A1				0.873*	0.547*
CYP3A2					0.742*

Level of significance: *P < 0.05

### Impact of facility, treatment-step and season on wastewater-induced CYP gene expression

**Fig 2:**
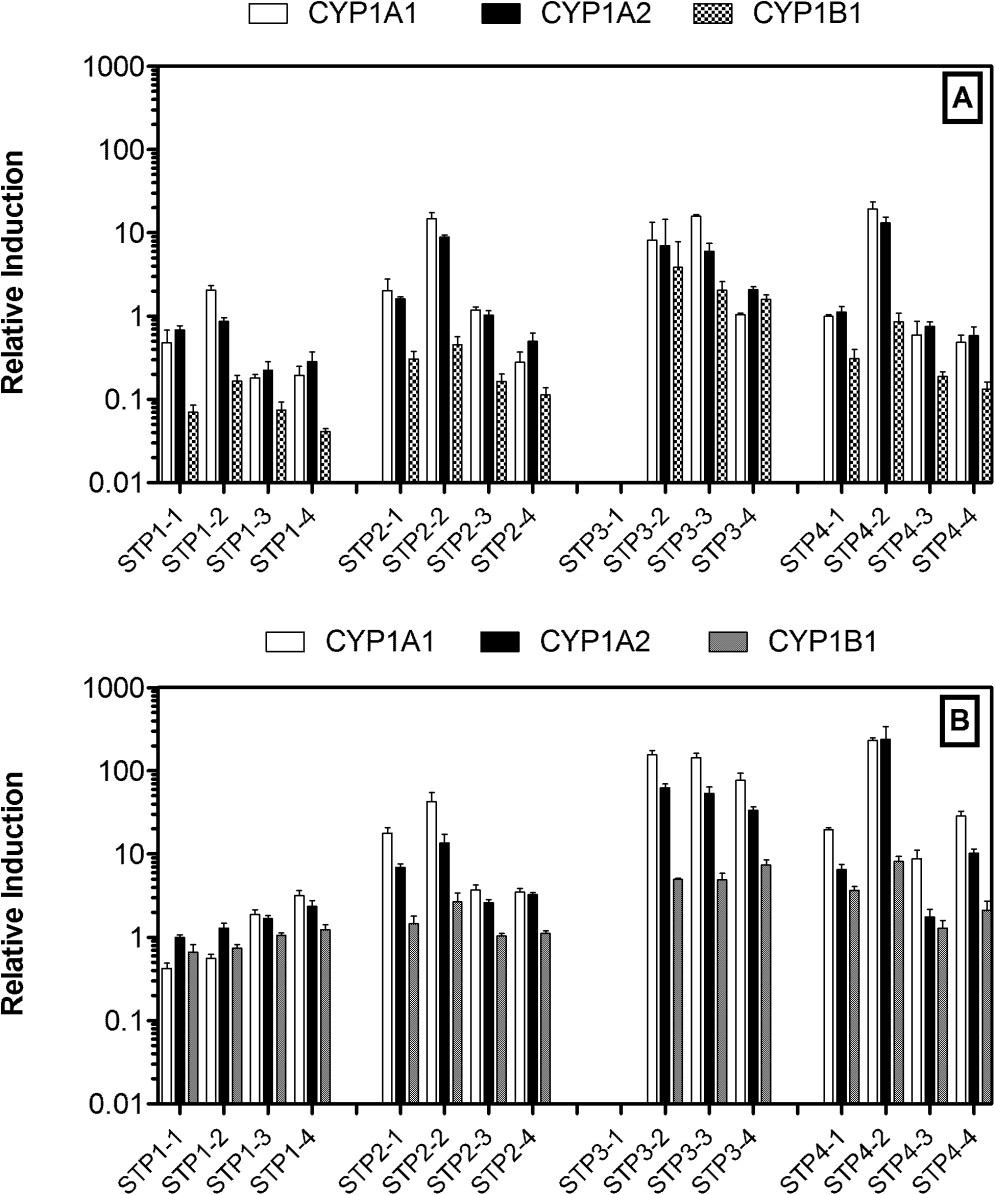
Comparison of CYP1 gene expression in rat hepatocytes exposed to wastewater extracts from STPs. A: Pre-monsoon, B: Monsoon. Data are reported as means ± SD (standard deviation). STPx-y: x is the type of STP, y is the treatment step [equalization (step 1), aeration (step 2), settling (step 3), and outlet (step 4)]. The data for STP3-1 were not available.

**Fig 3:**
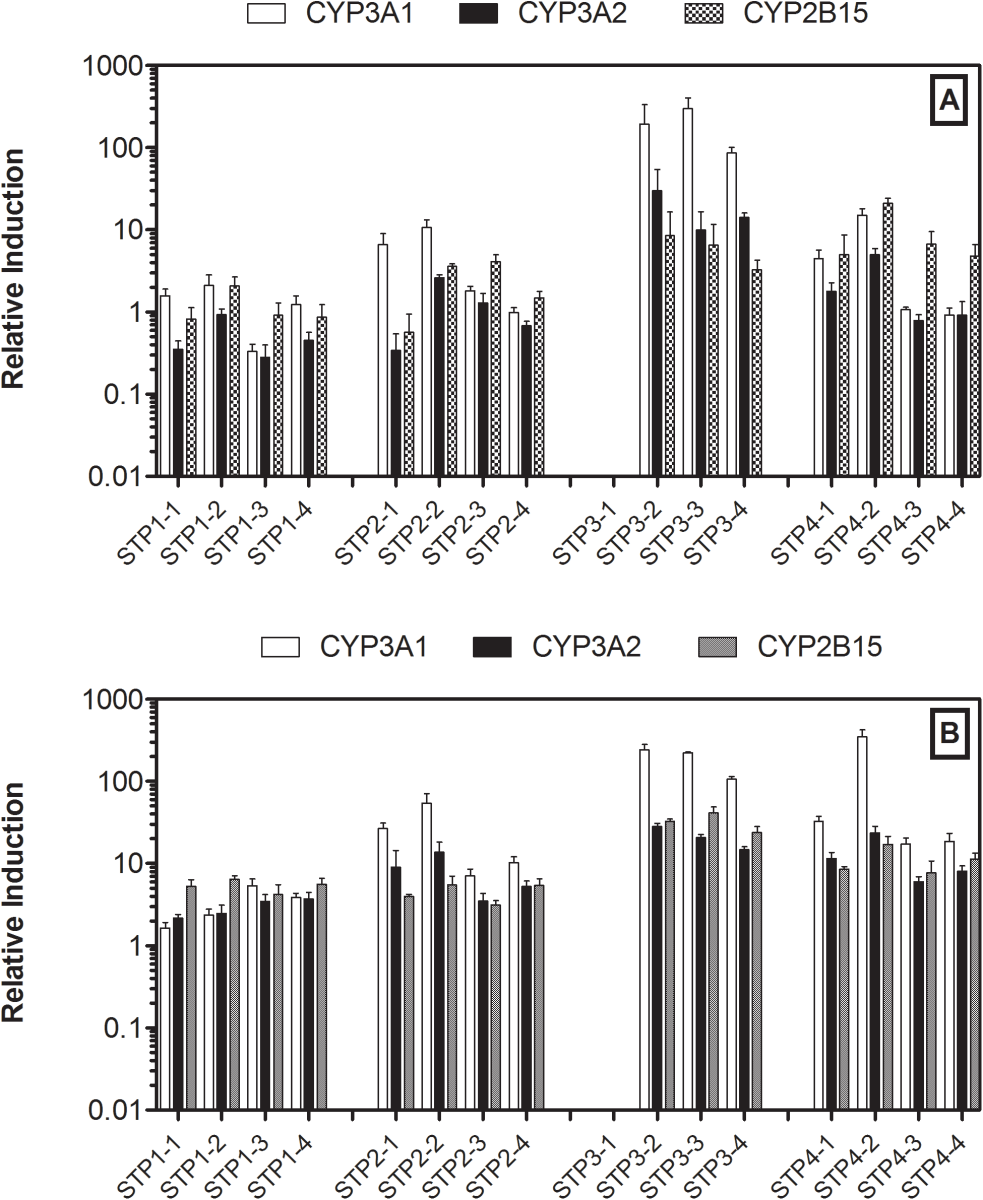
Comparison of CYP2B15 and CYP 3A gene expressions in rat hepatocytes exposed to wastewater extracts from STPs. A: Pre-monsoon; B: Monsoon. Data are reported as means ± SD (standard deviation). STPx-y: x is the type of STP; y is the treatment step [equalization (step 1), aeration (step 2), settling (step 3), and outlet (step 4)]. The data for STP3-1 were not available.

The procedural blank-normalized levels of expression of the CYP1A1, 1A2 and 1B1 genes induced by the STP extracts in the pre-monsoon and monsoon seasons are shown in Fig 2. The seasonal variations in the abilities of the wastewater extracts to induce expression of the CYP2B15, 3A1 and 3A2 genes in rat hepatocytes are shown in Fig 3. STP-3 did not have a step-1 sample; hence no data are available for this site. The data exhibited distinct patterns of wastewater-induced levels of gene expression between STPs, processing steps and seasons. In order to characterize the differential levels of gene expression induced by STP wastewater, the gene expression data were tested by a multiple regression analysis model. The associations between the expression level of each gene and the treatment facility, season, and treatment steps data as variables are given in Table 2.

**Table 2:** The estimated associations between gene expression potential and sewage treatment facility, season and treatment process in STP wastewater.

Gene	Parameter	Coefficient	Standard error	P-value
CYP1A1	Constant	0.269	0.6275	0.067
	STP-1	-2.208	0.685	0.005**
	STP-3	1.758	0.644	0.014*
	STP-4	0.355	0.606	0.565
	Monsoon	2.292	0.477	<0.001***
	Step 2	1.155	0.675	0.104
	Step 3	-0.244	0.675	0.722
	Step 4	-0.895	0.703	0.219
CYP1A2	Constant	0.164	0.508	0.749
	STP-1	-1.335	0.555	0.027*
	STP-3	1.496	0.522	0.010*
	STP-4	0.311	0.491	0.535
	Monsoon	1.661	0.387	<0.001***
	Step 2	1.235	0.547	0.036*
	Step 3	-0.362	0.547	0.516
	Step 4	-0.487	0.569	0.403
CYP1B1	Constant	-1.495	0.291	<0.001
	STP-1	-0.536	0.318	0.110
	STP-3	1.821	0.299	<0.001***
	STP-4	0.488	0.282	0.100
	Monsoon	1.846	0.222	<0.001***
	Step 2	0.484	0.314	0.140
	Step 3	-0.271	0.314	0.399
	Step 4	-0.300	0.326	0.371
CYP3A1	Constant	1.497	0.512	0.009
	STP-1	-1.554	0.559	0.012*
	STP-3	3.173	0.526	<0.001***
	STP-4	0.297	0.495	0.555
	Monsoon	1.496	0.390	0.001**
	Step 2	0.632	0.551	0.266
	Step 3	-0.530	0.551	0.349
	Step 4	-0.992	0.574	0.101
CYP3A2	Constant	-0.143	0.335	0.673
	STP-1	-0.669	0.366	0.084
	STP-3	1.878	0.344	<0.001***
	STP-4	0.509	0.324	0.133
	Monsoon	1.565	0.255	<0.001***
	Step 2	0.896	0.360	0.023*
	Step 3	-0.008	0.360	0.983
	Step 4	0.049	0.375	0.897
CYP2B15	Constant	-0.059	0.301	0.845
	STP-1	0.053	0.329	0.874
	STP-3	1.325	0.310	<0.001***
	STP-4	1.156	0.291	<0.001***
	Monsoon	1.073	0.229	<0.001***
	Step 2	1.007	0.324	0.006**
	Step 3	0.609	0.324	0.077
	Step 4	0.473	0.338	0.179

The explanatory variables, STP-2 (facility: relatively newly build and receives mixed domestic and hospital wastewater), pre-monsoon (season) and step 1 (process) were used as the reference levels. The asterisks shown to the P values shows the levels of statistical significance (* <0.05, **<0.01, ***<0.001) difference.

To evaluate the statistical significance of the effect of treatment facility on the ability of wastewater to induce gene expression, the STP-2 data were used as a reference. STP-1, which receives only domestic wastewater, exhibited a significantly lower ability to induce CYP1A1, 1A2 and 3A1 expressions at probability levels of P<0.01, P<0.05, P<0.05, respectively, compared to the STP-2 facility, which receives a mix of domestic and hospital wastewater. In contrast, STP-3, which exclusively receives hospital wastewater, demonstrated significantly higher induction capacity for all genes (P<0.05-P<0.001) compared to the STP-2 facility. STP-4 is a relatively old facility but receives a mix of wastewater from domestic and hospital sources, similar to STP-2, and had a significantly positive influence on CYP2B15 (P<0.001) expression similar to that of STP-3. Monsoon wastewater had significantly higher gene induction capacity than pre-monsoon wastewater (P<0.01 for CYP3A1, other genes P<0.001). Our multivariable regression analysis identified that the expressions of CYP1A2 (P<0.05), 2B15 (P<0.05) and 3A2 (P<0.01) were significantly greater in treatment step 2, where biological treatment by aeration takes place with activated sludge, compared to those in step 1 (equalization step). Nevertheless, no significant reduction of gene expression potency in wastewater was observed at the step 4 (outlet samples) compared to the step 1 (equalization) in these facilities.

### Association between gene expression potential and prevalence of resistant *E*.*coli* in STP wastewater

**Fig 4:**
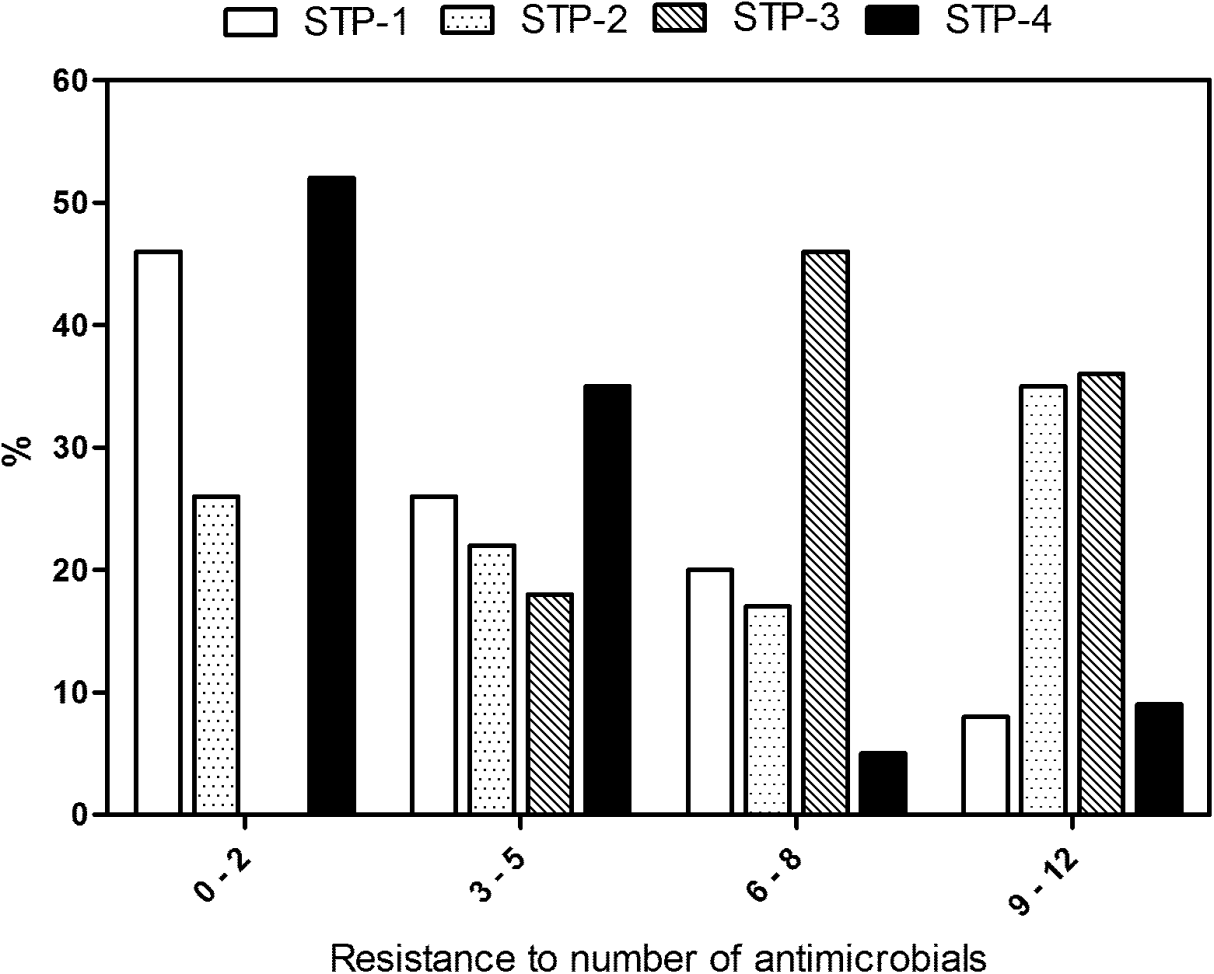
The number of drugs to which *E*. *coli* isolates from four different sewage treatment plants (STPs) in South India showed antimicrobial resistance. The X-axis indicates the number of resistant-antimicrobials of *E*. *coli* isolates. The Y-axis represents the percentage of the resistant *E*. *coli* isolates. Data for STP-1, -2 and -3 were retrieved from Akiba et al., 2015.

For the current study, antimicrobial susceptibility data to 12 antimicrobials of 226 *E*.*coli* isolates collected during the pre-monsoon and monsoon seasons from STPs were included to elucidate the associations between gene expression potential and the prevalence of drug-resistant *E*.*coli* in STP water. The distribution of the number of drugs to which the *E*. *coli* isolates showed resistance is given in Fig 4. About 72%, 48%, 18% and 86% of isolates exhibited resistance to less than 6 antimicrobials, whereas 28%, 52%, 82% and 14% of isolates showed resistance to more than 6 antimicrobials tested from the STP-1, -2, -3 and -4, respectively.

To evaluate the influence of CYP induction capacity on the dissemination of drug-resistant *E*.*coli* in the STP wastewater, we conducted a multivariable logistic regression analysis considering the influences of type of STP, treatment step and season as variables. Interestingly, the data showed that the prevalence of cefotaxime- and imipenem-resistance of *E*. *coli* was significantly correlated (P<0.05) with the CYP1B1 and 3A2 gene induction potency of the STP wastewater (Table 3). Moreover, CYP2B15 expression was significantly correlated with the existence of streptomycin-resistant (P<0.05) *E*.*coli* isolates in these 4 facilities. There were no significant correlations found between prevalence to other antimicrobials tested with *E*. *coli* isolates or gene induction potency (S2 Table, P>0.05). In addition, the data revealed that isolates in STP-4 showed significantly lower resistance to at least 7 antimicrobials tested compared to those in the STP-2 while monsoon season was negatively influenced on two antimicrobials (Table 3 and S2 Table). Moreover, no significant associations were found between the prevalence of *E*. *coli* with drug-resistance to any of the tested antimicrobials and the treatment steps in STPs (Table 3 and S2 Table).

**Table 3:** The estimated associations between resistance of *E*. *coli* isolates to an antimicrobial agent and gene expression potential with other confounding variables in STP wastewater.^1^

Antimicrobial	Parameter	Coefficient	Standard Error	P-value
Cefotaxime	Constant	-0.689	0.628	0.272
	CYP1B1	0.546	0.274	0.046*
	STP-1	-0.530	0.386	0.170
	STP-3	-0.460	0.571	0.420
	STP-4	-1.233	0.405	0.002*
	Monsoon	-0.624	0.513	0.224
	Step 2	-0.413	0.375	0.271
	Step 3	-0.066	0.379	0.863
	Step 4	0.038	0.355	0.915
Imipenem	Constant	-3.598	0.971	<0.001
	CYP3A2	1.321	0.575	0.022*
	STP-1	0.913	0.764	0.232
	STP-3	-1.694	1.076	0.115
	STP-4	-0.913	0.765	0.233
	Monsoon	-0.656	0.848	0.439
	Step 2	-1.013	0.777	0.192
	Step 3	-0.206	0.776	0.791
	Step 4	0.409	0.677	0.546
Streptomycin	Constant	0.198	0.312	0.525
	CYP2B15	0.613	0.271	0.023*
	STP-1	-0.562	0.358	0.116
	STP-3	-0.919	0.516	0.075
	STP-4	-1.169	0.512	0.022*
	Monsoon	-1.372	0.437	0.002**
	Step 2	-0.876	0.460	0.057
	Step 3	-0.751	0.421	0.074
	Step 4	-0.385	0.360	0.284

^1^Data shows if only significant association between an antimicrobial agent and gene expression was found. All other data are given in S2 Table. STP-2, pre-monsoon and Step 1, were assigned as the reference level for the variables of the sewage treatment facility, season and processing step, respectively. The asterisks shown to the P values shows the levels of statistical significance (* <0.05, **<0.01).

## Discussion

In this study we verified several well-known prototypical CYP450 gene inducers as positive controls to validate our *in vitro* gene expression bioassay of rat primary cultured hepatocytes integrated with a qRT-PCR method. Among the selected inducers, PB, DEX, PCN, RIF, OMP and TCPOBOP are an antiepileptic drug, an anti-inflammatory drug, a synthetic steroid, an antibiotic, a proton-pump inhibitor and a PB-like CYP-inducing agent, respectively. The AhR agonist β-NF is a chemopreventive agent while TCDD is a member of the POP family. The concentrations of all inducers tested in our study were similar to the concentrations that were previously employed in several *in vitro* assays studying receptor-regulated transcription factors without causing a substantial cell toxicity [30, 39–41]. The induction of the CYP1, 2B and 3A genes was mediated through AhR, CAR and PXR, respectively, and the crosstalk between CAR and PXR has been well documented (31, 42). Our CYP gene expression data were in good agreement with earlier studies in which rat cells were exposed to these inducers [39, 42, 43]. Much like the authors of theses previous reports, we observed that RIF, OMP and TCPOBOP showed weak or no effects on the gene expressions in rat hepatocytes studied here.

Although there was inconsistency in the step-wise gene expression potentials of STP wastewater observed in rat hepatocytes, it appeared that none of the treatment plants adequately detoxified the wastewater through the treatment processes used. Removal efficiencies of pollutants range according to contaminant characteristics and treatment processes. The treatment technology of wastewater in STP-1, -2 and -4 were comparable and consisted of activated sludge treatment, settling tank, with pressurized sand filtration and/or granular activated carbon (GAC) filtration before releasing treated water to the environment. The STP-4 did not have GAC filtration whereas STP-3 had a duel-media filter system where water is eventually discharged into an underground drainage. Previously, we reported the concentrations of chloramphenicol, trimethoprim, sulfamethoxazole, and ofloxacin in pre-monsoon wastewater samples collected from STP-1, -2, and -3 [13]. In addition, by following the same protocol, we analyzed these 4 antimicrobials in wastewater samples collected from STP-4. Chloramphenicol concentration was <10 ng/L in wastewater samples collected from all the sites. However, chloramphenicol was not detected in wastewater samples collected from STP-1. Trimethoprim concentrations at steps 1 and 4 were 285 and 125 ng/L, respectively, in wastewater samples collected from STP-2 and 36 and 4 ng/L, respectively, in wastewater samples collected from STP-4, suggesting that its concentration decreased by >50% due to adsorption on sludge particles. Trimethoprim was not detected in wastewater samples collected from STP-1, and its concentration at steps 2 and 4 was similar (43 ng/L) in wastewater samples collected from STP-3. In contrast, sulfamethoxazole concentration in wastewater samples collected from the outlets of STP-2 and STP-4 (633 and 91 ng/L, respectively) was higher than that in samples collected from step 1 of STP-2 and STP-4 (207and 26 ng/L, respectively). Deconjugation of conjugated pharmaceuticals, which occurs during wastewater treatment, may be a reason why higher levels of free pharmaceuticals were detected in the outlets of STPs than in incoming water. Ofloxacin concentration decreased from 579 to 368 ng/L (57% decrease) and from 2469 to 2158 ng/L (13% decrease) in wastewater samples collected from the outlets of STP-4 and STP-2, respectively, due to treatment. In contrast, ofloxacin concentration increased from <1.6 to 212 ng/L in wastewater samples collected from the outlet of STP-1 but remained unchanged (from 537 to 500 ng/L) in samples collected from the outlet of STP-3. These data indicated that input of pharmaceutical type, load, and operational performance of these treatment plants were dissimilar. Low chloramphenicol concentrations were correlated with the lower prevalence of chloramphenicol-resistant *E*. *coli* found in wastewater [13]. However, other antimicrobials examined in this study did not show any association with the prevalence of respective resistant *E*. *coli* in wastewater. In a previous study, dichloromethane extracted wastewater collected from a similar treatment plant in northern India demonstrated androgenic and other toxic effects in rats which may be elicited by nonylphenol, hexachlorobenzene and other natural androgens found in the samples [44]. The authors have noted that despite treatment and removal of considerable quantity of chemicals in inlet water, the effects of outlet water were still enough to pose endocrine-disrupting toxicity.

Our study design with hexane/dichloromethane extraction appears to extensively enrich non-to less-polar chemicals in the extracts. For instance, such less hydrophilic contaminants such as hydrocarbons and their halogenated derivatives were widely detected together with PPCPs in STP effluents and sewage sludge [25, 45–46]. Contaminant with high *K*_*ow*_ (octanol-water partition coefficients) tend to sorb onto the sludge particles and are removed with the sewage sludge [4]. Our samples were not filtered as we intended to elucidate potential total effects of wastewater in bulk and therefore considerable portion of chemicals could be derived from suspended particles. This was supported with our observations, where all step 2 (aeration tank) samples had visible amount of suspended particles, revealed significantly greater gene stimulation potencies compared to step 1 (Table 2).

Comprehensive monitoring studies of micropollutants and there toxicological effects in STP environments in India are not well documented. Hepatic gene expression data can be employed to gather preliminary information on gene-related drugs by accessing numerous online databases [47]. We attempted to use this approach to identify all the chemicals that are known to interact with the examined genes within the curated chemical-gene interaction data from the Comparative Toxicogenomics Database. There were 332, 233, 80, 34, 116 and 134 CAS-registered chemicals that were documented to have affected the rat (*Rattus norvegicus*) CYP1A1, 1A2, 1B1, 2B15, 3A1 and 3A2 gene response, respectively. Among them, 108 chemicals were involved in the regulation of all or more than the three targeted genes (S3 Table). The predicted chemicals which could be found in STP wastewater were similar to a number of chemical groups such as POPs, PAHs, pesticides and PPCPs previously detected in wastewater [25]. Since we did not intend to analyze wide range of chemical contaminants in the present study, based on the findings in the bioassay, probable candidates for the observed gene expression should subsequently be clarified.

Our data suggested that the monsoon season had a significant effect on the ability of wastewater to enhance gene induction compared to this ability of wastewater in the pre-monsoon season (Table 3). The monsoon rain can surge an extra load of chemical and biological constituents into the STPs, where the urban runoff can mix with the domestic/industrial effluents at the inlet. A positive correlation between antimicrobial resistance and rainfall has been reported in surface waters [33]. In contrast, while the treatment processes did not exhibit an influence on prevalence of ARB, the monsoon samples showed a marginally negative effect on the resistance of *E*.*coli* isolates against the antimicrobials streptomycin and kanamycin (Table 3 and S2 Table). The monsoon weather could be a cause of the substantial seasonal variance in drug usage, which in turn drives the seasonal increase in communicable diseases in certain areas in India [48].

Our results suggest that hospital wastewater (STP-3) had a significantly higher capacity for induction of all the CYP genes compared to the mixed-source STP effluents (Table 3). In contrast, the samples from STP-1, which exclusively receives domestic wastewater, had less impact on CYP1As and 3A1 gene induction potential compared to those in mixed-source effluents. A wide range of chemicals, including PPCPs, pesticides, steroids and carcinogens, can share CAR and PXR and regulate the induction of CYP3As [49]. For example, being a CAR agonists, non-dioxin like polychlorinated biphenyls induced CYP2B, in addition to stimulation of the PXR to some degree to induce CYP3A1 in rat hepatocytes [41]. Although there are pharmacological differences in the regulation of CYP3A expression among species, it is widely accepted that CYP3As are the most abundant hepatic phase I enzyme that metabolizes most of marketed drugs [42, 50]. Likewise, presence of a wide range of nonpolar to mid-polar compounds, such as dioxins, PCBs, PAHs and a wide range of PPCPs in these STPs were predicted from our gene-chemical interaction data. It was also suggested that hydrophobic pharmaceutical compounds in the hospital wastewater which were released via feces could pose a higher environmental risk than some of the other compounds present [51]. Therefore, the greater induction of CYP genes may reflect the presence of many such compounds in the hospital wastewater (STP-3) compared to domestic effluents (STP-1). The CYP1A1 induction via AhR activation of wastewater tested in our study showed an induction capacity comparable to that by the treatment with approximately 2.72 mg/L of β-NF, while the CYP3A1 induction via PXR activation showed an induction capacity similar to that by treatment of cells with approximately 232 mg/L of PB or 7.86 mg/L of DXM. Additionally, we found that all genes studied had significant positive correlations in their inductions (Table 2). Hence, our gene expression data indicate the presence of individual contaminants at greater concentrations and/or synergistic effects of many ligands due to the complex nature of chemicals in the treated wastewater. In an earlier study, extremely high pharmaceutical concentrations exceeding 16 mg/L in wastewater from treated effluents originated from drug manufacturers in Hyderabad, India, was reported [52]. Fish exposed to a 0.5% solution of the same wastewater exhibited the expression of hepatic CYP1A genes together with other physiological dysfunctions [53]. Although we have reported few antimicrobials in STP wastewaters which ofloxacin at a concentration up to 2.5 μg/L, detailed monitoring study should be carried out to identify and quantify individual toxicants (13).

We have previously found that water treatment in the STP-1, -2 and -3 slightly decreases the total viable counts (TVCs) of bacteria and total coliforms (TCs) in pre-monsoon wastewater samples. Nevertheless, samples collected from the outlets of STP-1, STP-2, and STP-3 still contained 4.0, 4.7, and 6.5 log_10_ CFU/mL of TVCs and 3.8, 3.8, and 6.2 log_10_ CFU/mL of TCs [13]. Similarly, TVC values of wastewater samples collected from step 1 and outlet of STP-4 were 6.8 and 5.2 log_10_ CFU/mL, respectively, while their TC values were 5.9 and 4.1 log_10_ CFU/mL, respectively. These data indicated that treated water from all the 4 STPs contained >3.7 log_10_ CFU/mL of TVCs or TCs. In this study, 1.5–2.3 log_10_ CFU/mL of TVC and TC were reduced in STP-1, STP-2 and STP-4 during the treatment process, while elimination of bacterial count in STP-3 was inadequate throughout the treatment process (outlet > 6.2 log_10_ CFU/mL). Collectively, these results suggested an ineffective treatment of the wastewater in these STPs.

The *E*. *coli* isolates from STP-3 demonstrated resistance to most of the antimicrobials tested. New data collected from STP-4, which receives a mixture of domestic and hospital wastewater, exhibited fewer isolates (14% of the total) exhibiting resistance to over 6 antimicrobials (Fig 4). Therefore, the impact from hospital wastewater on the distribution of ARB at STP-4 was probably lowest during the time of sampling at two sampling seasons. STP-2 receives wastewater on a priority basis and when it operates in full capacity, STP-4 acquires the remaining wastewater for treatment. Therefore, the quality and quantity of chemicals and microorganisms in wastewater in STP-2 and -4 could be diverse. These results were further supported by the multivariate logistic regression analysis, which showed that the isolates from STP-4 had significantly lower resistance to at least seven of the antimicrobials tested (S2 Table).

In order to assess the impact of the release of antimicrobial-resistant organisms into the environment, as well as to devise strategies for its mitigation, previous reports have discussed such related issues as anthropogenic activities, hospital effluents, environmental contaminant and nutrient levels and several geographical and climatic parameters [33, 54]. Although we did not analyze some of them, it is expected that the effects of contaminants in STP wastewater can influence the selectivity of drug resistant bacteria. In consequence, we attempted to integrate transcription factor-mediated gene expression in mammalian cells as a new independent variable to elucidate impact on antimicrobials resistance in *E*. *coli*. Where the information of contaminants occurs in wastewater was limited, effect-based data would be appropriated for the hazard assessment. Our analysis illustrated that the gene induction potency in wastewater had a significant relationship with *E*. *coli* resistance to two β-lactam antimicrobials. Among them, cefotaxime is a 3^rd^ generation cephalosporin, while imipenem is a carbapenem-type antimicrobial that plays an important role in the treatment of many multi drug-resistant bacteria. The use of β-lactams seems to be increasing in India [55]. On the other hand, streptomycin-resistance was also positively associated with CYP2B15 induction. This antibiotic seems to be widely used in developing countries because it is inexpensive and readily available. The data for the gene expression potency in the present study were from rat hepatocytes; however, it should be noted that such responses to chemicals differ considerably among animal species (31). The gene alteration capacity of the above-mentioned antimicrobials in rat *in vitro* system is not well known, and further assessment to identify potential antimicrobial-gene interactions should be performed in the future. Nevertheless, it has been widely accepted that there is still a knowledge gap of recognizing the factors and mechanism that drive antimicrobial resistance in wastewater (9). In the present study, we focused on *E*. *coli*. However, other genera of bacteria such as *Enterococcus*, *Acinetobacter*, and *Staphylococcus*, which are known to carry drug resistance genes, in wastewater should be assessed in future studies. We believe that exploiting gene expression potency data would provide additional information of synergistic chemical effects in STP wastewater for understanding the dissemination of ARBs.

In conclusion, this study demonstrates that a bioassay based on the primary culturing of hepatocytes could be a useful tool to reveal the biological effects of the complex chemical mixtures within the wastewater at STPs. Our data also indicated that, even though the treatment the wastewaters at these four STPs may have reduced the levels of some chemicals, the abilities of the wastewater to induce the expression of various genes still remained stable throughout the treatment steps. Hospital wastewater could strongly influence the toxic effects mediated through transcription factor-related gene expression. The bioassay data may also provide vital information on the occurrence and properties of chemicals in STPs. Our data suggest that the gene-induction capacities of chemicals could be further used to clarify the proliferation of antimicrobial-resistant bacteria in wastewater. Further studies are needed to establish the identity of the active compounds in the STPs in India.

## Supporting Information

S1 Table Primer sequences for real-time qRT-PCR. (XLSX)

S2 Table Estimated associations between resistance of *E*. *Coli* isolates to an antimicrobial agent and gene expression potential with other confounding variables in STP wastewater.^1^
^1^Estimated results were from a multivariable logistic regression analysis model. STP-2, pre-monsoon and Step 1, were assigned as the reference level for the variables of the facility, season and processing step, respectively. The asterisks shown to the P values shows the levels of statistical significance (* <0.05, **<0.01). NA^2^: Estimate value and statistical significance for coefficients of these variables were not available because of quasi-complete separation issue (Chloramphenicol).
(XLSX)

S3 Table Chemicals which show interactions with more than three examined CYP genes in rats. Data were retrieved from the curated chemical-gene interaction data from the Comparative Toxicogenomics Database.
(XLSX)
